# Fighting force and experience combine to determine contest success in a warlike mammal

**DOI:** 10.1073/pnas.2119176119

**Published:** 2022-06-14

**Authors:** P. A. Green, Faye J. Thompson, Michael A. Cant

**Affiliations:** ^a^Centre for Ecology and Conservation, College of Life and Environmental Sciences, University of Exeter, Penryn, Cornwall TR10 9FE, United Kingdom;; ^b^Deutsch Primate Center, University of Goettingen, Goettingen 37707, Germany

**Keywords:** animal behavior, social evolution, animal contests, intergroup contests

## Abstract

Conflicts between social groups or “intergroup contests” are proposed to play a major role in the evolution of cooperation and social organization in humans and some nonhuman animal societies. In humans, success in warfare and other collective conflicts depends on both fighting group size and the presence and actions of key individuals, such as leaders or talismanic warriors. Understanding the determinants of intergroup contest success in other warlike animals may help to reveal the role of these contests in social evolution. Using 19 y of data on intergroup encounters in a particularly violent social mammal, the banded mongoose (*Mungos mungo*), we show that two factors, the number of adult males and the age of the oldest male (the “senior” male), have the strongest impacts on the probability of group victory. The advantage conferred by senior males appears to stem from their fighting experience. However, the galvanizing effect of senior males declines as they grow old until, at very advanced ages, senior males become a liability rather than an asset and can be evicted. As in human conflict, strength in numbers and the experience of key individuals combine to determine intergroup contest success in this animal society. We discuss how selection arising from intergroup contests may explain a suite of features of individual life history and social organization, including male eviction, sex-assortative alloparental care, and adult sex ratio.

## Significance

Intergroup conflict has been proposed as a major influence in social evolution. Understanding how intergroup contests exert selection on group living requires determining what properties of groups and their members drive contest success. We analyzed 19 y of data on intergroup fighting in wild banded mongooses to disentangle the factors that determine victory. Two factors, the number of males in the group and the age of the oldest “senior” male, most strongly influence the probability of victory. Senior males may be a benefit because of their disproportionate fighting experience. As in human societies, strength in numbers and the presence of key individuals are critical for success in violent intergroup contests, perhaps influencing selection on individual life history and social behavior.

From raiding termites (1) to warring humans (2), many cooperative animal societies engage in aggressive intergroup fights, or contests, over resources. These intergroup contests have been proposed as a major force favoring the evolution of within-group altruism and between-group hostility in humans and other animals (3–5). However, what determines success in collective fights? In historical human battles, strength in numbers was often a major determinant of conflict success (6, 7). Effective leadership or the presence of “key individuals” is also crucial in human intergroup contests ranging from small raiding parties to battles between vast armies (8, 9), as charismatic warriors or gifted leaders can help smaller fighting units to prevail against larger ones (9–12). Similarly, in nonhuman animals, group size, or sometimes the number of members of a certain subgroup (e.g., males), are important factors in intergroup contest success (13–17), and key individuals, such as leaders, may also improve group fighting ability. Key individuals contribute to group success in contexts like foraging, patrolling, and defense (8, 18, 19). However, few studies have examined whether key individuals enhance collective fighting ability in nonhuman animals, that is, whether certain individuals have disproportionate effects on intergroup contest success (15, 20).

We examine how group and individual factors combine to determine success in intergroup conflicts in a model wild mammal species, the banded mongoose. The aim is to understand the determinants of collective fighting ability or group resource holding potential (RHP) (21). In classic dyadic animal contest theory, RHP has a profound influence on conflict behavior and selection for weaponry and variation in life history allocation (22–24). Experimentally, RHP is measured by proxy as the properties of individuals that most strongly predict competitive success (25). For dyadic contests, proxies of RHP might evolve, at least in part, for their role in combat. For example, claw grasping strength is a proxy of RHP in many crab species, and species that use their claws more intensely in combat possess stronger, more mechanically efficient claws (26). Testing among multiple potential RHP proxies for intergroup contests (e.g., refs. 15, 17, and 27) might reveal properties of groups or group members (e.g., life history traits or individual and collective decision-making) that evolve under selection for their role in contests.

## Results

We studied a wild population of banded mongooses in Uganda that is typically composed of 10 to 12 social groups, each with ∼20 adults plus offspring. Banded mongoose groups contain multiple breeding males and females. Females give birth synchronously (usually on the same night), and males help in both babysitting newborn pups at the den and in escorting older pups as they learn to forage. New groups form most often by the eviction of same-sex cohorts (usually females) from existing groups, which are joined by dispersing groups of the opposite sex (reviewed in ref. 28).

**Fig. 1.**
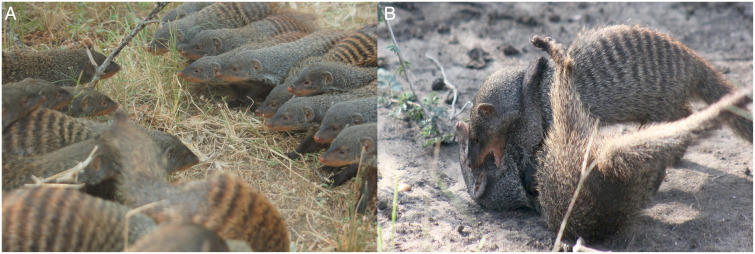
Intergroup contests in banded mongooses. (*A*) Groups often start contests by forming “battle lines” before breaking into (*B*) physical fighting that involves biting and wrestling. Image credits: (*A*) Harry Marshall, University of Roehampton, London, UK; (*B*) Dave Seager, University of Cambridge, Cambridge, UK.

As in other cooperatively living taxa, banded mongooses commonly engage in intergroup contests over access to territory and the resources therein, including food; groups may also compete over access to extragroup mating opportunities (Fig. 1) (29, 30). At our study site, the average rate of intergroup contests varies widely over time and is particularly high when new groups are trying to establish a territory and when females in groups are in estrus. Estimates of the mean number of contests per group over the course of the study have ranged from 0.7 (29) to 4.1 per month (30). These values are on par with other social living species (*SI Appendix*, Table S1). Banded mongoose intergroup contests are highly aggressive and can result in injury or even death of combatants. This species is among a handful of social mammals (including lions, wolves, chimpanzees, and humans) for which intergroup conflict accounts for 10% or more of adult deaths of known cause (31–33). The median annual rate of adult mortality that can be directly attributed to intergroup conflict is 0.4% (31); this rate is much higher than that of a closely related herpestid, meerkats (median = 0.0%, mean = 0.2%), and is similar to the median rates in an analysis of chimpanzees (0.3%), hunter-gatherers (0.2%), and subsistence farmer societies (0.6%) (data are from refs. 31 and 32). While all group members participate in intergroup contests, males are more aggressive than females (30) and are much more likely to die during or as a direct result of contests (31). Subordinate males (defined as those who did not mate guard a female in the prior breeding cycle) more readily approach out-group competitors than do dominant males (males who did mate guard a female in the prior breeding cycle) (30). Thus, previous behavioral observations suggest that group members participate differentially in contests, leading to alternative hypotheses for which members might most strongly influence contest outcomes.

Intergroup contest RHP might be driven not only by which group members participate in contests but also by properties of those group members themselves. Individual body size is often an RHP proxy in dyadic contests (25, 34, 35), and the body size of group members may influence intergroup contest outcomes (36). Group member age may also be an RHP proxy; in dyadic contests, age and fighting experience are thought to influence contest behaviors and possibly, outcomes (37–39). Further, while the number of group members is clearly an aggregate property of the group, group member weight and age may function at a group level (e.g., groups with on-average heavier or older group members may win) or at the level of the individual (e.g., groups with the heaviest or oldest members may be most successful).

We asked how intergroup contest RHP in banded mongooses was approximated by group- and individual-level properties by testing which properties best predict contest success (25). From observations of 90 naturally occurring contests with known winner and loser groups, in which we had full data on individual sex, age, and weight, we calculated relative (focal group minus rival group) metrics of the number of adult group members, member weight, and member age for three classes of group members: all group members, males only, and subordinate males only. While females were considered in the “all group members” class, we did not use a “females only” class because evidence suggests females contribute much less than males to intergroup fighting (above and refs. 30 and 31). For weight and age variables, we calculated both mean values (e.g., the relative mean weight of all subordinate males) and maximum values (e.g., the relative age of the oldest male) between groups. We built 12 generalized linear mixed models (GLMMs) that predicted whether focal groups won or lost from relevant combinations of these predictors and compared the explanatory power of the predictors in these models using an information-theoretic approach (*Materials and Methods*).

**Fig. 2.**
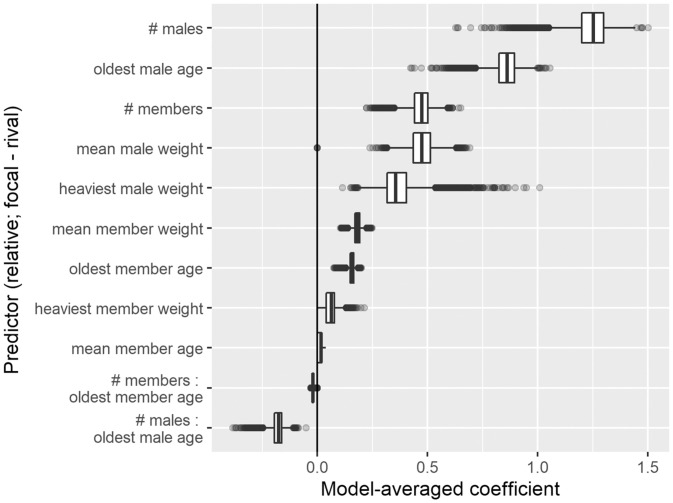
Relative number of males and relative oldest male age best predicted contest success. Model-averaged coefficients (*x* axis; measuring the effect of each predictor on contest outcomes) of predictors (*y* axis) from the top model set. All predictors are relative (focal group values minus rival group values). Box plots show medians (thick lines), interquartile ranges (IQRs; box edges), 1.5× IQRs (whiskers), and outliers (points) from 10,000 iterations of weight data imputation and model comparisons as detailed in *Materials and Methods*.

The relative number of males and the relative age of the oldest male were the best predictors of intergroup contest success (Fig. 2 and *SI Appendix*, Table S3). Other properties were included in the top model set and were positively correlated with contest success, including relative mean male weight, which was in the best-fit model (*SI Appendix*, Table S2). However, the number of males and oldest male age were in four of the top five models (*SI Appendix*, Table S2), and the 95% CIs of metrics of variable importance (*Materials and Methods*) for the number of males and oldest male age did not overlap with those of any other properties (Fig. 2 and *SI Appendix*, Table S3), showing their heightened importance to contest success and, therefore, their importance as proxies of RHP. The number of males and oldest male age had an equal likelihood of occurring in the best-fit model, but the number of males had a stronger effect on contest outcomes (*SI Appendix*, Table S3).

In a follow-up analysis, we refit the best-fit model using a Bayesian approach that constrained the focal and rival group random effects to be equal and opposite. This approach is recommended when testing the effects of relative (e.g., focal minus rival) variables on contest outcomes; because focal and rival identities are arbitrarily assigned, the variances of the focal and rival random effects should be equal to each other, and their correlation should be equal to −1 (e.g., refs. 27 and 40). The fixed effect estimates from this Bayesian model were qualitatively similar to those from our information-theoretic approach (*Materials and Methods* and *SI Appendix*, Table S4).

The role of the number of males as an RHP proxy may relate to sexual dimorphism relevant to the physical fighting common in banded mongoose intergroup contests. In the population as a whole, males were heavier than females (male weight mean ± SD [*N* males] = 1,440.1 ± 276.0 g [1,651]; female weight mean ± SD [*N* females] = 1,293.0 ± 262.5 g [1,430]; linear mixed model [LMM] sex [male] *β* ± SE = 108.7 ± 11.9, *χ*^2^_1_ = 80.2, *P* < 0.01) (*SI Appendix*, Table S8), and males had wider heads than females (male head width mean ± SD [*N* males] = 40.0 ± 6.1 mm [1,357]; female head width mean ± SD [*N* females] = 38.7 ± 5.5 mm [1,094]; LMM sex [male] *β* ± SE = 0.8 ± 0.2, *χ*^2^_1_ = 10.4, *P* < 0.01) (*SI Appendix*, Table S9). Both traits may be important in physically attacking rivals (e.g., wrestling, biting) (Fig. 1*B* and Movie S1). For example, head width is correlated with bite force across mammalian taxa (41), and bite force predicts contest success in some systems (42, 43). In contrast to the number of males, the positive impact of oldest male age was not likely related to physical properties; oldest males did not also have the heaviest weights (proportion of oldest males that were also the heaviest males in their groups = 13/66 males, 19.7%) or widest heads (proportion of oldest males that were also males with widest heads in their groups = 27/67 males, 40.3%) of males in their groups.

**Fig. 3.**
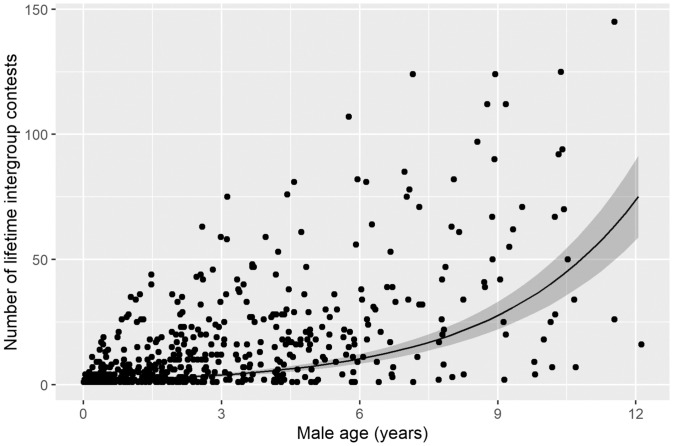
Age is related to contest experience. The number of intergroup contests a male participates in over its lifetime increases with age. Points represent individual observations, the line represents the prediction from the GLMM (*Materials and Methods*), and the shaded area shows the SE around the model prediction.

We hypothesized that the influence of having the oldest males in either competing group (hereafter, “senior” males) might arise because senior males have the most experience in intergroup conflicts. Although likely not the only important factor, this experience might, in some way, confer an advantage on the performance of their group. Supporting this hypothesis, we found that, for males across all intergroup contests (*n* = 853 males in 319 contests), there was a significant and positive correlation between male age and the number of contests in which a male participated over his life (GLMM; scaled age estimate ± SE = 0.8 ± 0.0, *χ*^2^_1_ = 507.7, *P* < 0.01) (Fig. 3 and *SI Appendix*, Table S10).

To explore other potential reasons why senior males might confer such an advantage on group fighting success, we examined whether the oldest male might have other same-aged male group mates fighting alongside him and whether senior males were behaviorally dominant in their own group. We compared senior males in groups that won while having a senior male (“winning groups,” 21 unique males represented 95 times across 66 contests) with senior males in groups that lost despite having a senior male (“losing groups,” 14 unique males represented 26 times across 24 contests). In a minority (21%) of 90 contests, there were either two (7 of 90) or three (12 of 90) senior males of equal age in the same group (total *N* contests = 90, total *N* senior males = 121, median *N* senior males per group = 1, range = 1 to 3). These same-age senior male group mates were more common in winning groups than in losing groups (winning groups proportion of same-age male group mates: 29/95 = 0.3, losing groups: 2/26 = 0.1, binomial test *P* < 0.01), suggesting that these cases where there were two or three same-aged males may have contributed to the strength of the senior male effect. However, we note that only relative oldest male age, not relative average male age, was a top predictor of contest success (Fig. 2 and *SI Appendix*, Table S3). Additionally, subsetting our dataset to only those contests in which there was one senior male (*n* = 71 contests) leads to nearly identical estimate values from the best-fit model (*SI Appendix*, Table S15). This finding suggests that, while additional senior males may play a role, the age of the oldest male is a major determinant of contest outcomes. Turning to the role of behavioral dominance, at the time of the contest, senior males in winning groups were not more likely to be dominant (i.e., to have mate guarded a female in the prior breeding cycle) than senior males in losing groups (winning groups proportion of dominant senior males: 26/95 = 0.3, losing groups: 7/26 = 0.3, binomial test *P* = 0.9). They were also not more likely to have been dominant at any point prior to the contest than senior males in losing groups; all senior males had been dominant before the contest. Thus, behavioral dominance does not explain the impact of senior males on contest success.

**Fig. 4.**
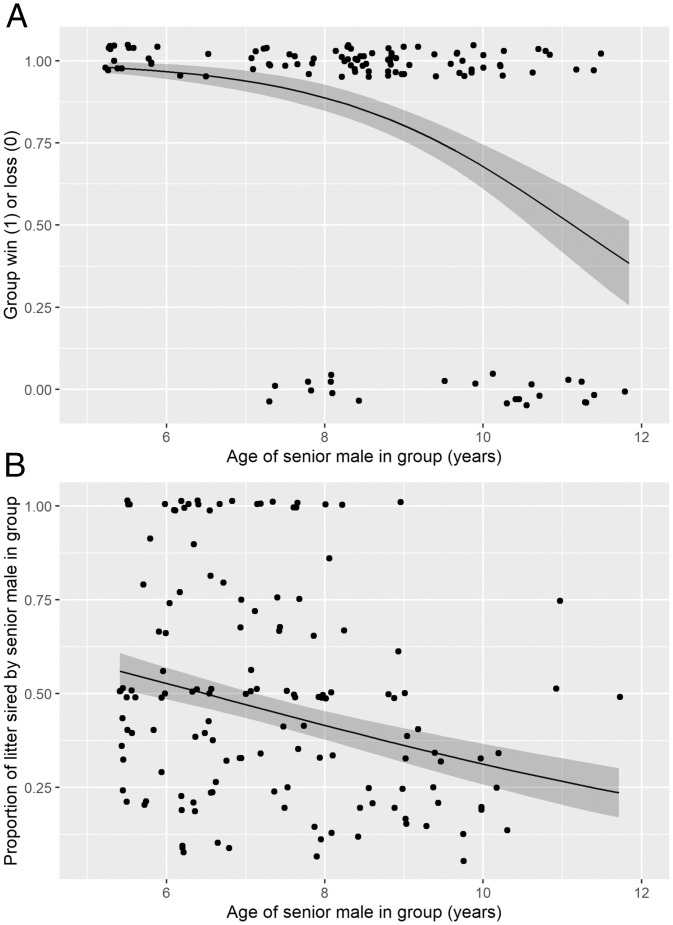
Senior males lose (*A*) intergroup and (*B*) within-group competitive ability with age. In *A*, the dataset includes all senior (i.e., oldest) males from groups that either won while having a senior male (*y* axis = 1) or lost despite having a senior male (*y* axis = 0; *n* = 23 unique males across 121 contests). The line shows the prediction from the GLMM, and the shaded area shows the SE. In *B*, the dataset includes 133 litters sired by 46 unique males. Each point shows the proportion of the litter sired by the senior males in the group (*y* axis) against the age of those senior males (*x* axis). Solid line shows the prediction from the GLMM, and the shaded area shows the SE. In both *A* and *B*, point location is jittered for clarity.

Although senior males improved group success in intergroup contests, this advantage declined with age, potentially suggesting a trade-off between the benefits of experience and the costs of senescence of competitive ability. Senior males in winning groups were, on average, nearly 2 y younger than those in losing groups (winning group senior males mean age ± SD = 8.3 ± 1.7 y; losing group = 10.0 ± 1.6 y). As senior male age increased, the likelihood of intergroup contest success decreased (GLMM; scaled age estimate ± SE = −1.2 ± 0.3, *χ*^2^_1_ = 11.3, *P* < 0.01) (Fig. 4*A* and *SI Appendix*, Table S11). In fact, this model predicted that senior males older than 11 y were a liability rather than a benefit to group success in contests (Fig. 4*A*). Older, less competitive males were also at heightened risk of being evicted from their group; the likelihood of male eviction increased with male age (GLMM; scaled age estimate = 0.5 ± 0.1, *χ*^2^_1_ = 15.7, *P* < 0.01) (*SI Appendix*, Table S12). Finally, another indicator of the decline in competitive ability in older senior males is that they showed a decrease in their ability to compete for paternity of pups within their own group (GLMM; scaled age estimate = −0.3 ± 0.1, *χ*^2^_1_ = 9.1, *P* < 0.01) (Fig. 4*B* and *SI Appendix*, Table S13), although they were still relatively successful when accounting for extragroup paternity (GLMM; scaled age estimate = −0.2 ± 0.1, *χ*^2^_1_ = 2.2, *P* = 0.1) (*SI Appendix*, Table S14; *SI Appendix* has more details). Combined, these findings reflect senescence of competitive ability in older senior males, with potential consequences of eviction from the group.

## Discussion

Proxies of RHP, or fighting ability, reveal what properties determine contest success (21) and lend insights to how selection acts in the context of competition (e.g., refs. 26 and 44). Our analyses were designed to reveal which of many group- and individual-level properties of banded mongoose groups have the greatest influence on success in intergroup contests. Of these factors, two stand out: the relative number of adult males and the relative age of the oldest male in each group.

The first of these major predictors, the relative number of adult males, fits with what we know about sex differences in contributions to intergroup aggression in this species. Although females play a major role in the initiation of conflict (31), our experiments and observations suggest they rarely engage in physical combat. Previous experiments show that males are the first to approach intruders in a territory and are more aggressive toward intruders than females are (30), while long-term data indicate that adult males are much more likely than adult females to die as a result of intergroup violence (31). Thus, it seems likely that the number of males is an important determinant of the strength of the collective fighting force in this species. Across other species, including humans, males play a disproportionate role in intergroup fighting (e.g., refs. 2, 31, 45, and 46). However, this is by no means universal; both sexes contribute more equally to intergroup contests in other nonhuman species, including certain primates (e.g., common marmosets, red-tailed monkeys, gibbons, rhesus macaques) (a meta-analysis is in ref. 13), meerkats (27), and green woodhoopoes (47).

In dyadic contests, the functional hypothesis for RHP posits that proxies of RHP should relate to fighting styles. For example, where contests involve physical fighting, proxies of RHP should be related to physical strength, whereas contests without physical fighting (e.g., that involve mostly noncontact signaling) might find endurance-related properties, such as fat stores, to be more important to outcomes (34, 48). Banded mongoose contests often involve chasing, scratching, wrestling, and biting (Fig. 1*B* and Movie S1). Males have, on average, heavier bodies and larger heads than females (*Results*), which could provide an advantage during these physical fights (greater body size and head size improve contest success in many animal taxa, including in many mammals) (e.g., refs. 25, 34, 35, 49, and 50). Compared with within-group contests, intergroup conflict is likely to exert strong selection on traits relevant to physical fighting in banded mongooses because, unlike male–male aggression that occurs in the context of within-group mating competition, intergroup aggression commonly results in injury and even death (31). The functional hypothesis can explain why the relative number of males per se, rather than the relative total group size, is a particularly important determinant of victory during banded mongoose intergroup contests.

More surprising was our finding that the relative age of a specific group member, the oldest male in each group, had a major impact on the likelihood of the group winning the contest. We currently do not have the type of detailed observational data necessary to reveal precisely why the oldest males in groups should have such a prominent sway over the outcome. However, our long-term data suggest that the advantage conferred on the group by senior males is linked to contest experience rather than physical attributes. Oldest males are not typically the largest or heaviest in the group (*Results*), but, on average, they have participated in the greatest number of contests (Fig. 3). Moreover, this experience effect may be compounded in successful groups; groups that won while having a senior male were more likely to have at least one additional senior male (i.e., one or two same-age male group mates) in their group compared with groups that lost despite having a senior male. Similar results were found in gray wolves, in which groups with a greater number of members older than the prime reproductive age were more likely to win intergroup contests (15). In addition to experience on its own, age might have a suite of other impacts on individual and group fighting ability. In dyadic contests, age can increase the motivation to be aggressive and gain resources from contests (39) and to engage in potentially lethal violence (51). Furthermore, fighting experience (which may increase with age) may help an individual better assess its ability relative to the population (37) and/or may improve fighting technique and skill relevant to winning (38). In the kind of collective battles among close-knit groups that we observe in banded mongooses, the presence of particularly experienced, skilled, or fearless individuals could have a cascading social influence on the fighting performance of the collective. The importance of key individuals to group fighting success is well known in the context of human conflict (8, 12, 52), but there is very little information on the potential galvanizing effects of key individuals in intergroup conflict among nonhuman animals (20).

The importance of males in general, and senior males in particular, to intergroup contest success may explain aspects of social behavior and life history in this system, such as patterns of helping, eviction, and longevity. In banded mongooses, pups receive one-to-one alloparental care from both male and female adult helpers, called escorts. Male pups receive more care from escorts, on average, than female pups (53). Moreover, male adults preferentially escort male offspring, thus investing in individuals that will become future cocombatants in intergroup fights (53). Outside of escorting relationships, adult males are less likely than females to be evicted from the group (54), and on average, adult males live longer than females (28). As a result of these sex differences in care, eviction, and mortality, the adult sex ratio within groups is strongly male biased, with ∼1.6 adult males to every adult female (28). We suggest that these patterns of 1) greater care for male pups, 2) reduced eviction of male adults, 3) longer male life span, and 4) male-biased sex ratio may have evolved under selection for the importance of males and senior males in intergroup contest success. That is, selection arising from intergroup conflict may explain patterns of social behavior, social organization, demography, and individual life history in banded mongooses. Intergroup conflict could potentially shape key features of the societies of other mammals, particularly those in which intergroup conflict is a major source of mortality (and thus, a major selective pressure), including wolves (33), lions (55), chimpanzees (32), and humans (32).

Finally, our results illustrate how the value and contributions of group members can vary systematically across their lifetimes as a consequence of age-linked changes in their cultural and physical influence. Senior male banded mongooses may contribute prowess or steadfastness in the face of battle, but eventually, these benefits appear to be offset by the limitations and frailties of old age. There is evidence of similar contrasting social impacts of age on sociocultural vs. physical influence of key individuals in other social mammals. In killer whales, elephants, and humans, grandmothers confer substantial benefits on their offspring and grandoffspring (56–61). However, one recent human study suggests that, at very advanced ages, grandmothers start to inflict inclusive fitness costs rather than benefits (59), potentially accelerating selection for senescence. In banded mongoose groups, older senior males become less of an asset in intergroup contests, and older males are at increased risk of eviction (*Results*). This finding, together with previous data on female evictions (62), may suggest that banded mongoose groups are more likely to evict individuals that inflict a net cost on the rest of the group.

The concept of RHP—properties predicting competitive success—is fundamental to the study of dyadic contests (21, 25), and recent studies have begun to explicitly consider how RHP proxies might evolve under the selective pressure of conflicts (26, 44). However, despite widespread interest in how warfare influences evolution in animal societies (3, 4, 20, 63, 64), explicit tests among multiple potential RHP proxies have been rare for intergroup contests. Future tests of RHP in intergroup contests may reveal the behavioral and tactical pathways to success in warfare while illuminating how selection in the context of competition acts on group member behavior, morphology, and life history as well as on group demography.

## Materials and Methods

### Ethical Note.

All research procedures received prior approval from the Uganda Wildlife Authority, the Uganda National Council for Science and Technology, and the Ethical Review Committee of the University of Exeter. All procedures adhere to the Association for the Study of Animal Behaviour / Animal Behavior Society Guidelines for Animals in Behavioral Research and Teaching.

### Study Population.

This study was conducted on a wild population of banded mongooses on and around the Mweya Peninsula in Uganda (0°12′S, 29°54′E). Data were collected using daily observations conducted between February 2000 and April 2019. All individuals in the population are uniquely identifiable by shave patterns on their back and pit tags under the skin of the nape of their neck (TAG-P-122IJ; Wyre Micro Design). Up to two members of each group are fitted with a radio collar (Sirtrack), allowing the group to be located daily. Further details can be found in ref. 28.

### Data Collection.

All data used in this study were from naturally occurring intergroup contests (*n* = 598). Following ref. 29, we defined an intergroup contest as any occasion in which two groups sighted each other and responded by emitting screeching calls, chasing, and/or fighting. We use this broad definition because while contests are always aggressive, the density of bushes and cover at our study site means that it is difficult to accurately determine whether there has been physical contact between groups. We only analyzed data for contests in which we observed a clear winner and clear loser and for which each group had at least one male and one subordinate male (*n* = 268). Winning and losing groups were identified through observations, such as which group retreated from the location of the contest (losing groups), made by a dedicated field team with over 70 y of combined experience. We did not include contests with “draw” outcomes in our dataset because the analysis required a binomial (win/lose) response. For each contest, we collected data on group membership, member age, and member weight.

Group membership data included the number of males and females greater than 6 mo old (29). Males were further designated as either subordinate or dominant at the time of the contest. Dominant males were those that mate-guarded estrus females during the most recent breeding period within 180 d before the contest; males who did not mate guard in this time were considered subordinate (30). Individual age data were calculated as the date of the intergroup contest minus the date the individual was born. All females within a group give birth synchronously (65), meaning all pups within a litter have the same birth date. Although pups are initially raised in underground dens, we are confident that birth dates are accurate to 24 h because groups are visited daily and there are obvious, visible changes in female size and weight that occur immediately after birth (65). Some individuals (242 of 3,913) were adult immigrants to the population; these individuals were assumed to be 2 y old on the date of their immigration (no senior males in contests were immigrants). Individuals are weighed at regular intervals (which vary depending on the group’s habituation to the experimenters) either by being trained to step on a portable electronic scale (Kern & Sohn 440-53N; Wolflabs) for a food reward or after being captured (Tomahawk Live Trap Co.), anesthetized using isoflourane, and placed on a scale. We only used weights for individuals weighed within 120 d before the contest (median duration between contest date and weighing date nearest to contest date = 5 d, range = 0 to 120 d) and for which we were confident the weight data were as accurate as possible. Weight data were highly repeatable within individuals; using the rpt function in R (66) on a dataset of 10,172 weight samples from 568 individuals weighed at least twice within 120 d of an intergroup contest (*N* contests = 218), the mean repeatability estimate was 0.9 (2.5, 97.5% percentiles = 0.9, 0.9; permuted *P* value median < 0.01 [2.5, 97.5% percentiles = 0.0, 0.0]).

We were missing contest weight data for 22.1% of individuals in the population. At least one individual in either competing group was missing weight data in 71.8% of groups on the date of the contest. We used an imputation approach to account for much of this missing weight data while not unduly influencing our analyses (*Weight Data Imputation*).

### Statistical Analysis.

All analyses were conducted using R version 4.0.2 (67).

### Weight Data Imputation.

For groups in which 20% or less of group members were missing weight data on the date of the intergroup contest (97 unique individuals from 122 contests in focal groups; 99 unique individuals from 104 contests in rival groups), we took advantage of the history of weight data within each group to impute missing weight values. Imputation approaches are an appropriate way to account for missing data without unduly affecting parameter estimates (68) and have been used in other studies of long-term populations in which missing data occur (56, 69). For each individual that was missing weight data, we identified all individuals in the same group and of the same sex (and for females, the same pregnancy status) that were within 120 d of age of the individual (60 d younger to 60 d older). We built a truncated normal distribution (the rtruncnorm function in the truncnorm package) (70) from the weight values of these same-group, same-sex, near-age individuals and randomly sampled one value from this distribution. This value was imputed for the individual’s missing weight value. To confirm that this approach did not significantly alter the weight relationships within each group, we built growth curves (the drm function in the drc package) (71) predicting weight from age (72) for each group without imputed data and with imputed data. These curves fit three parameters to the asymptotic weight–age growth curve using a self-starting function (the fct = AR.3 argument in the drm function): the lower limit of the growth curve, its asymptote, and the slope of its increase. The parameter estimates from these curves did not differ between the two datasets, showing that our imputation approach did not alter within-group weight relationships (*SI Appendix*, Table S5). The imputation procedure resulted in a dataset in which at least one individual was missing weight data in 45.8% of groups (compared with 71.8% preimputation; see above). The final dataset included full data on both focal and rival groups (11 groups total) from 90 contests occurring between November 2004 and March 2019. Below, we detail how we used repeated sampling to account for the random nature of this imputation.

### Hypothesis Testing: Model Comparison Approach.

We used a model comparison approach following ref. 73 and references therein to test among alternative hypotheses for which group and group member properties best predicted contest success and therefore, which properties best approximate RHP. We built 12 global GLMMs (lme4 package) (74), each one representing a hypothesis for which properties influence contest success (*SI Appendix* has all model forms). Models included relative values calculated as focal group value minus rival group value (e.g., the relative number of members for a focal group with 20 members and a rival group with 8 members was 12). Group focal/rival identity was randomly determined by 1) what group the observers were following when a contest occurred or 2) using the sample function in R. The predictors in all models were scaled with unit variance using the scale function. All models also included random effects of focal group identification and rival group identification (data showing the proportion of contests won by each group are reported in *SI Appendix*, Table S6). Finally, all models had binomial error structures with logit link functions. We ensured good model fit and a lack of collinearity in predictor variables by plotting model residuals, checking correlations between predictor variables, and checking variance inflation factors with the check_collinearity function in the performance package (75).

We used the dredge function in the MuMIn package (76) to calculate Akaike Information Criterion corrected for small sample size (AICc) scores for models including all subsets of predictors within each of the 12 global models. We combined all of these models, removed duplicated models (i.e., models with the exact same combination of terms), and retained only those models with ΔAICc < 6 from the best-fit model (i.e., the model with the lowest AICc score). Finally, we implemented a nesting rule (77) such that any model that had a worse fit than a simpler model that included the same terms was thrown out. In this way, we saved only models that included the fewest terms and had the best fits. Our top model set consisted of all nested subsets of the 12 global models that had ΔAICc < 6 from the best-fit model.

To account for variation in the random sampling used to impute weight data, we repeated our weight imputation and model comparison approach 10,000 times. Each iteration, we repeated the random sampling of weight data and the model comparison approach. We then removed models from the top model set that occurred in <50% (i.e., <5,000) of the iterations, as these models may have been rare occurrences more influenced by the random sampling approach instead of real trends in the data. Models that did not meet this 50% cutoff were rare and/or relatively poor fits to the data (*SI Appendix*, Table S7). After filtering, we calculated the 2.5, 50 (median), and 97.5% percentiles of the AICc score; model likelihood; and model weights for each model in the top model set. Model likelihood $\left(l_{i}\right)$ shows the relative likelihood of model i given the data calculated as $l_{i}=e^{-0.5\times \Delta \mathrm{AIC}c_{i}}$. Model weight $\left(w_{i}\right)$ shows the probability of each model i given the data calculated as $w_{i}=l_{i}/\sum_{j=1}^{R}l_{j}$ (78).

We also calculated the 2.5, 50 (median), and 97.5% percentiles of the model-averaged coefficients and variable importance scores for predictors in this filtered top model set. Model-averaged coefficients give the parameter estimates for each predictor in each model weighted by that model’s weight in the top model set. These are calculated as $\bar{\beta }=\;\sum_{i=1}^{R}w_{i}{\hat{\beta }}_{i}$, where ${\hat{\beta }}_{i}$ is the coefficient of the predictor in model i and $w_{i}$ is the weight of model i. ${\hat{\beta }}_{i}$ is zero when the predictor is not in the model. Predictors with stronger effects on contest outcomes and that occur in more better-fitting models will have higher model-averaged coefficients (79, 80). Variable importance quantifies the likelihood that a given predictor appears in the best-fit model of the top model set and is simply the sum of the model weights (w) for each model in which the predictor occurs. Terms with higher variable importance values are more likely to occur in the top model set (79).

### Bayesian Analysis.

In analyses of the effect of relative (focal minus rival) predictors on contest outcomes, only one observation (one outcome) is recorded per contest between focal and rival group. This observation (focal win or focal lose) depends on the arbitrary designation of the focal group; the outcome is one if the focal group won the contest but zero if the rival group won the contest. To account for this arbitrary designation in these analyses, the effects of focal and rival identity on contest outcomes—that is, their random effect variances—should be equal, and their correlation opposite (e.g., refs. 27, 40, and 81 have further justification). We conducted a Bayesian analysis that fit these constraints to our model and showed that this change did not affect our results (*SI Appendix*).

### Follow-Up Analyses.

We used several follow-up analyses to interrogate the effects of male membership and oldest male age on contest outcomes.

To understand the effect of male membership, we built LMMs (lme4 package) predicting weight and separately, head width from sex. For weight, we used all data on all adults (>6 mo age) in the population, excluding data from pregnant females (results were qualitatively similar when including pregnant females). Head width data were taken from a separate dataset in which head width was measured when individuals were captured and anesthetized (samples sizes are in the text). The LMMs predicted either weight or head width from sex, and both included a random effect of individual identity. We compared the fit of each model (weight or head width) with a model that did not include the fixed effect of sex using a likelihood ratio test in the drop1 function. We also used these weight and head width data to ask whether senior males were also the males with the greatest weights or widest heads in their groups on the date of the contest. We calculated the proportion of senior males that also had the heaviest weight (or greatest head width) of all members of their group within 120 d of the contest.

To explore the effect of senior male age, we first correlated the number of contests males had ever been involved in with male age. For each male in the focal and rival groups of our contest dataset (*n* = 319 contests), we calculated 1) the total number of contests in which they were involved in the dataset and 2) the maximum age recorded for them in the dataset. We built a GLMM (glmmTMB package) (82) with a Poisson error structure and log link function predicting the total number of contests from maximum age. This model also had an observation-level random effect to account for overdispersion. We used the DHARMa package (83) to confirm good model fit. We used the drop1 function to compare the likelihood ratio of this model with a model without the fixed effect of maximum age.

Next, we combined data on all individuals from focal and rival groups. We subset this dataset to include only the senior males from groups that won while having the senior male(s) (i.e., the oldest males in focal groups that won when focal–rival maximum male age was >0 and the oldest males in rival groups that won when focal–rival maximum male age was <0). We also created a dataset of the senior males from groups that lost despite having the senior male(s) (i.e., the oldest males in focal groups where the focal groups lost when relative male age was >0 and the oldest males in rival groups where the rival groups lost when relative male age was <0). We compared these datasets (hereafter, the winning dataset and the losing dataset, respectively).

Using these datasets, we tested whether senior males in winning groups were more likely to have same-age group mates than senior males in losing groups. From both the winning and losing datasets, we quantified the number of duplicate age values (i.e., the number of male group mates that were the same age on the date of the contest as the senior males). We used a binomial test (binom.test function) to compare the proportion of same-age group mates in the winning dataset with that from the losing dataset. The null hypothesis of this test was that these proportions were the same (i.e., that the senior males from winning groups were not more likely than the senior males from losing groups to have same-age group mates).

To further test the impact of multiple senior males on contest success, we subset our original dataset (*n* = 90) to include only contests in which there was one senior male (*n* = 71). We fit the best-fit model from *SI Appendix*, Table S2 to this dataset and compared the estimate values for all predictor variables (*SI Appendix*, Table S15).

To assess the role of senior male dominance in contest success, we tested whether senior males from the winning dataset were more likely to be dominant. From the winning dataset, we calculated the proportion of senior males that were dominant (i.e., that were observed mate guarding within 180 d of the contest). We calculated the same proportion of dominant senior males from the losing dataset. We compared these proportions using a binomial test; the null hypothesis was that the senior males in the winning dataset were not more likely to be dominant than the senior males in the losing dataset.

We also tested whether the senior males from groups that won while having the senior male were more likely to have been dominant at any point prior to the contest. From the winning and losing datasets, we calculated the total number of times each senior male had ever been dominant in its life. We did not statistically test this comparison as all senior males in both datasets had previously been dominant.

Next, we asked how the age of senior males influenced their groups’ intergroup contest success. After combining the winning and losing datasets, we built a GLMM (lme4 package) with a binomial error structure and logit link function predicting whether the group won or lost the contest from male age. This model had a random effect of individual identification to account for multiple measures from the same male (e.g., where the male was the senior male in the group when it both won and lost a contest). We used the drop1 function to compare the likelihood ratio of the model with the age fixed effect with a model without this effect.

To understand the relationship between senior male age and paternity, we tested how the proportion of pups sired by the oldest males in groups correlated with the age of those oldest males. *Results* has a reporting of the methods for this analysis.

Finally, we tested how the rate of male eviction was predicted by male age. We used an approach modified from ref. 62 but with an expanded dataset and an analysis focused to our specific question of how age predicts eviction likelihood. We identified 57 eviction events in which 148 unique males were evicted from their groups (total *N* of all unique males in groups = 543). We built a GLMM with a binomial error structure and logit link function predicting the probability of a male being evicted from the group (1 = yes, 0 = no) from male age. Male identification, group identification, and the identification of the eviction event were random effects. We used the drop1 function to compare the likelihood ratio of this model with a model without the fixed effect of male age.

## Supplementary Material

Supplementary File

Supplementary File

## Data Availability

Data (.csv files) and analysis code (.R files) are publicly accessible in FigShare (https://doi.org/10.6084/m9.figshare.14815173.v1).
