# Supplementary material for: Fighting force and experience combine to determine contest success in a warlike mammal
Source: Proc Natl Acad Sci U S A. 2022 Jun 14;119(25):e2119176119. doi: 10.1073/pnas.2119176119 (PMC9231503; doi:10.1073/pnas.2119176119)
Supplement: Supplementary File [file pnas.2119176119.sapp.pdf]

Supplemental Information for

Fighting force and experience combine to determine contest success in a warlike mammal

P.A. Green, Faye J. Thompson, Michael A. Cant

Corresponding authors: P.A. Green; pagreen@ucsb.edu

Michael A. Cant; m.a.cant@exter.ac.uk

This DOCX file includes:

- List of Global Models
- Bayesian Analysis Methods
- Paternity Analysis Methods
- Tables S1 to S15
- Supplementary Information References
- Movie S1 legend

Other supplementary materials for this manuscript include the following:

- Movie S1

LIST OF GLOBAL MODELS

All fixed effects are relative (focal – rival) and scaled with unit variance

RE_only: P(focal group win) ~ 1 + (1|focal group ID) + (1|rival group ID)

Models 1-4: models focusing on traits of all members

Mod.1: P(focal group win)~ # members + mean member weight + mean member age + # members : mean member weight + # members : mean member age + mean member weight : mean member age + (1|focal group ID) + (1|rival group ID)

Mod.2: P(focal group win)~ # members + max member weight + max member age + # members : max member weight + # members : max member age + max member weight : max member age + (1|focal group ID) + (1|rival group ID)

Mod.3: P(focal group win)~ # members + max member weight + mean member age + # members : max member weight + # members : mean member age + max member weight : mean member age + (1|focal group ID) + (1|rival group ID)

Mod. 4: P(focal group win)~ # members + mean member weight + max member age + # members : mean member weight + # members : max member age + mean member weight : max member weight + (1|focal group ID) + (1|rival group ID)

Models 5-8: models focusing on traits of males only

Mod.5: P(focal group win)~ # males + mean male weight + mean male age + # males : mean male weight + # males : mean male age + mean male weight : mean male age + (1|focal group ID) + (1|rival group ID)

Mod.6: P(focal group win)~ # males + max male weight + max male age + # males : max male weight + # males : max male age + male male weight : max male age + (1|focal group ID) + (1|rival group ID)

Mod.7: P(focal group win)~ # males + max male weight + mean male age + # males : max male weight + # males : mean male age + max male weight : mean male age + (1|focal group ID) + (1|rival group ID)

Mod.8: P(focal group win)~ # males + mean male weight + max male age + # males : mean male weight + # males : max male age + mean male weight : max male age + (1|focal group ID) + (1|rival group ID)

Models 9-12: models focusing on traits of subordinate males only

Mod.9: P(focal group win) ~ # subordinate males + mean subordinate male weight + mean subordinate male age + # subordinate males : mean subordinate male weight + # subordinate males : mean subordinate male age + mean subordinate male weight : mean subordinate male age + (1|focal group ID) + (1|rival group ID)

Mod.10: P(focal group win) ~ # subordinate males + max subordinate male weight + max subordinate male age + # subordinate males : max subordinate male weight + # subordinate males : max subordinate male age + max subordinate male weight : max subordinate male age + (1|focal group ID) + (1|rival group ID)

Mod.11: P(focal group win) ~ # subordinate males + max subordinate male weight + mean subordinate male age + # subordinate males : max subordinate male weight + # subordinate males : mean subordinate male age + max subordinate male weight : mean subordinate male age + (1|focal group ID) + (1|rival group ID)

Mod.12: P(focal group win) ~ # subordinate males + mean subordinate male weight + max subordinate male age + # subordinate males : mean subordinate male weight + # subordinate males : max subordinate male age + mean subordinate male weight : max subordinate male age + (1|focal group ID) + (1|rival group ID)

BAYESIAN ANALYSIS METHODS AND RESULTS

As stated in the Materials and Methods, our model fitting approach did not account for the fact that the random effect variances of focal and rival group identity should be equal and opposite (see, e.g., 1–3 for further justification). We were unable to impose these constraints in our maximum likelihood-based model comparison approach, because no model comparison framework (e.g., AIC) is appropriate for models in which random effects can be constrained. Therefore, we tested whether the estimates from the predictors of our best-fit model (see Table S2) using the model comparison approach were similar to those of same model when it was fit using the constraints described above.

We fit this constrained model using the MCMCglmm package in R (4) as a Bradley-Terry model (5) with random effects, broadly following the methods used in (1). This model had uninformative parameter-expanded priors and ran for 1,050,000 iterations, where the first 50,000 iterations were discarded and samples were saved every 250 iterations. We ensured good model fit by checking that autocorrelation was low among consecutive thinned observations and fixed effects, and that Heidelberg and Geweke diagnostic values and plots met expectations for good model fit. We fit this model to 2,000 of the 10,0000 iterations of random weight sampling described in the Materials and Methods, and each time we saved the fixed effect estimates from the model. In Table S4, we compare the 2.5%, 50% (median), and 97.5% percentiles of the fixed effect estimates of each predictor of the constrained model to the same percentiles of the estimates from the maximum likelihood model used in our information-theoretic approach. The 95% confidence intervals of each fixed effect in the Bayesian models are either close to (for number of males) or overlap with (for maximum male age and mean male weight) those of the maximum likelihood models (Table S4). Therefore, although our maximum likelihood approach

could not incorporate the fully appropriate random error structure, we are confident our parameter estimates are accurate.

PATERNITY ANALYSES

Within groups, males compete to mate-guard estrus females and sire pups. Previous research shows that the oldest males in groups sire the most pups (6). To further probe the potential for senescence of competitive ability in senior males, we tested whether older males showed a decrease in their ability to compete over paternity within groups. Using previously-constructed paternity data for our population (7, 8), we identified 1720 pups in 388 litters sired by 250 males**.** We subset this paternity dataset to only male sires that were at least as old as the youngest senior males we identified from our contest dataset and that were also the oldest in their group at the time of the pups' births (342 pups, 133 litters, 46 oldest males). We calculated, for each litter in each group, the proportion of pups sired by the oldest males in the group (in 7 of 133 litters, two or more senior males of the same age sired pups). We built a GLMM with a binomial error structure and logit link function in which the proportion of pups in each litter sired by the oldest males in the group was predicted by the age of the oldest males in the group. Litter ID, group ID, and sire ID were all random effects. We used the drop1 function to compare the likelihood ratio of this model to a model without the fixed effect of oldest male age. We compared this model to one that incorporated extra-group paternity. In this analysis, we calculated, for each litter in each group, the proportion of pups sired by the oldest males in any group in the population, not just in the group to which the litter was born (370 pups, 145 litters, 50 oldest males; in 12 of 145 litters, two or more oldest males of the same age sired pups). We built a GLMM of the same structure as that described above, and conducted the same statistical test as described above.

The within-group paternity analysis showed a negative relationship between the age of the oldest males in groups and the proportion of within-group pups that those males sired (GLMM; scaled age estimate = -0.3 ± 0.1, χ^2_1 = 9.1, P < 0.01; Figure 4B; Table S13); that is, older males were less able to secure within-group mating success. However, when we incorporated data on extra-group paternity—i.e., when we predicted the proportion of pups in litters that were sired by any oldest males in the population, not just those within the group—the relationship was not different from zero (GLMM; scaled age estimate = -0.2 ± 0.1, χ^2_1 = 2.2, P = 0.1; Table S14). This suggests that, as senior males age, they are less able to compete within the group for paternity, but they were still relatively successful when accounting for extra-group paternity.

TABLE S1. LITERATURE SURVEY OF INTERGROUP CONTEST RATES

We surveyed the literature for studies of intergroup contests in which data was either reported on, or from which we could calculate, the per-group rate of intergroup contests (N contests per month). Columns show the common and scientific names for each species, the number of groups studied, the per-group rate of intergroup contests, notes on how the per-group rate was calculated, and references. In the "N contests / month / group" column, asterisks indicate studies that detailed the standard error or standard deviation as well as the mean rate. We only report the mean rate for clarity with other studies. While results from banded mongooses (the focus of the present study) are presented first, other studies are reported in alphabetical order of species name.

**Common name**	**Species name**	**N groups**	**N contests/ month / group**	**Notes**	**Reference(s)**
Banded mongoose	*Mungos mungo*	10-12	0.73*		(9)
Banded mongoose	*Mungos mungo*	12	4.10	Calculated from N contests / hr. Includes variation during reproductive cycle	(10)
Black howler monkey	*Alouatta pigra*	5	0.8	N contests / N months / N groups	(11)
Gray wolves	*Canis lupus*	33	0.02	N contests / N months / N groups	(12)
White-faced capuchins	*Cebus capucinus*	5	0.32	N contests / N months / N groups	(13)
Capuchin monkey	*Cebus capucinus*	6	0.57	N contests / N months / N groups	(14)
Blue monkey	*Cercopithecus mitis*	6	3.6	N contests / N months / N groups	(15)
Vervet monkeys	*Chlorocebus aethiops pygerythrus*	4	2.36	N contests / N months / N groups	(16, 17)
Guerezas	*Colobus guereza*	8	1.02	N contests / N months / N groups	(18)
Mountain gorillas	*Gorilla beringei beringei*	1	0.78*		(19)

Mountain gorillas	*Gorilla beringei beringei*	14	0.21	N contests / N months / N groups	(20)
Lowland gorillas	*Gorilla gorilla gorilla*	3	0.73*		(21)
Japanese macaques (Yakushima)	*Macaca fuscata*	7	2.01*	N contests / hour / group	(22)
Japanese macaques (Kinkazan)	*Macaca fuscata*	3	1.44*	N contests / hour / group	(22)
Barbaray Macaques	*Macaca sylvanus*	1	0.5	N contests / N months / N groups	(23)
Chimpanzees	*Pan troglodytes*	1	0.67	N contests / N months / N groups	(24)
Baboons	*Papio cynocephalus*	5	0.4	N contests / N months / N groups	(25)
Green woodhoopoes	*Phoeniculus purpureus*	12	0.61	N contests per group / N months	(26)
Verraux's sifakas	*Propithecus verreauxi*	8	0.75*		(27)
Verraux's sifakas	*Propithecus verreauxi*	5	1.27	N contests / N months / N groups	(28)
Verraux's sifakas	*Propithecus verreauxi*	5	0.34	N contests / N months / N groups	(29)
Tufted capuchin monkey	*Sapajus nigritus*	4	1.44	N contests / N months / N groups	(11)
Meerkat	*Suricata suricatta*	36	0.09	N contests / N months / N groups	(1)

TABLE S2. MODEL AICc SCORES

Results of the analysis of which properties best predicted intergroup contest success (N = 90 contests among 11 groups). Models presented are those in the top model set: within 6 AICc of the best-fit model and that occurred in at least 5,000 of 10,000 iterations of the random weight imputation process (see Materials and Methods). Null model (including only random effects of focal and rival group identity) AICc = 102.0 (ΔAICc from median value of best-fit model = 21.2). N models shows the number of times each model was saved in the top model set (maximum = 10,000). Median AICc, 2.5%, and 97.5% percentiles are shown. ΔAICc shows each model's median AICc minus the best-fitting model's median AICc. Both model likelihood (l_i) and model weight (w_i) are presented with median, 2.5%, and 97.5% quantiles. Instances where median AICc, 2.5%, and 97.5% quantiles are equal occur when predictors do not include weight (as weights are the only values that are changed across iterations). Rows are ranked in order of increasing median AICc score.

**Predictors**	**N models**	**median AICc (2.5%, 97.5%)**	**Δmedian AICc**	**median l_i (2.5%, 97.5%)**	**median w_i (2.5%, 97.5%)**
# males, max male age, mean male weight	10000	80.826 (80.294, 81.328)	0	1.000 (1.000, 1.000)	0.345 (0.267, 0.414)
# males, max male age, max male weight, # males : max male age	9999	82.547 (81.434, 83.452)	1.721	0.426 (0.279, 0.707)	0.146 (0.104, 0.208)
# members, max member age, mean member weight	10000	82.631 (82.247, 82.999)	1.805	0.405 (0.339, 0.483)	0.140 (0.109, 0.163)
# males, max male age, max male weight	10000	82.945 (82.038, 83.665)	2.119	0.347 (0.247, 0.508)	0.120 (0.091, 0.150)

# males, max male age, # males : max male age	10000	84.635 (84.635, 84.635)	3.809	0.149 (0.114, 0.191)	0.052 (0.037, 0.062)
# members, max member age, max member weight	9998	84.700 (83.434, 85.886)	3.874	0.144 (0.077, 0.269)	0.050 (0.029, 0.078)
# males, max male age	10000	84.867 (84.867, 84.867)	4.041	0.133 (0.101, 0.170)	0.046 (0.033, 0.055)
# members, mean member weight	10000	85.354 (84.922, 85.758)	4.528	0.104 (0.086, 0.125)	0.036 (0.028, 0.042)
# members, mean member age, max member weight	7046	86.268 (85.164, 86.935)	5.442	0.066 (0.051, 0.115)	0.022 (0.017, 0.034)
# members, max member age, # members : max member age	9620	86.338 (86.338, 86.338)	5.512	0.064 (0.052, 0.082)	0.022 (0.017, 0.027)

TABLE S3. MODEL-AVERAGED COEFFICIENTS AND VARIABLE IMPORTANCE

Model-averaged coefficients for each predictor are calculated as $\bar{\beta} = \sum_{i=1}^{R} w_i \hat{\beta}_i$, where $\hat{\beta}_i$ is the coefficient of the predictor in model i and w_i is the weight of model i. $\hat{\beta}_i$ is zero when the predictor is not in the model. Variable importance is the sum of the model weights (w) for each model in which the predictor occurs. Rows are ranked in decreasing order of median model-averaged coefficient.

**Predictor**	**Median model-averaged coefficient (2.5%, 97.5%)**	**Median variable importance (2.5%, 97.5%)**
# males	1.253 (1.054, 1.364)	0.716 (0.602, 0.779)
max male age	0.861 (0.722, 0.944)	0.716 (0.602, 0.779)
# members	0.475 (0.373, 0.552)	0.265 (0.211, 0.307)
mean male weight	0.475 (0.362, 0.587)	0.345 (0.267, 0.414)
max male weight	0.356 (0.240, 0.540)	0.267 (0.196, 0.357)
mean member weight	0.181 (0.142, 0.214)	0.175 (0.137, 0.205)
max member age	0.157 (0.126, 0.179)	0.212 (0.173, 0.242)
max member weight	0.063 (0.023, 0.111)	0.070 (0.029, 0.110)
mean member age	0.017 (0.000, 0.026)	0.020 (0.000, 0.032)
# members : max member age	-0.020 (-0.025, 0.000)	0.022 (0.000, 0.027)
# males : max male age	-0.176 (-0.243, -0.134)	0.199 (0.156, 0.254)

TABLE S4. BAYESIAN AND MAXIMUM LIKELIHOOD MODEL COMPARISON

Predictors are those in the best-fit model as in Table S2.

**Predictor**	**Bayesian estimate median (2.5%, 97.5%)**	**Maximum likelihood estimate median (2.5%, 97.5%)**
# males	2.16 (1.90, 2.41)	1.76 (1.74, 1.78)
max male age	1.19 (1.00, 1.39)	1.22 (1.20, 1.24)
mean male weight	1.58 (1.24, 1.93)	1.38 (1.31, 1.45)

TABLE S5. COMPARISON OF GROWTH MODELS BEFORE AND AFTER WEIGHT DATA IMPUTATION

Columns show group ID for focal and rival groups, as well as the 95% CI and maximum and minimum values of the C (lower limit), D (asymptote), and E (slope of increase) parameters from growth curves fitted by the drm function. 95% CI values are calculated from original (i.e., non-imputed) data, while minimum and maximum values are calculated from imputed data. Because the minimum and maximum values for each parameter in the imputed data do not extend beyond their respective 95% CIs in the original data, the imputation did not significantly alter model fits. The final two columns show the number of individuals with weight data in the original and imputed datasets.

	ID	2.5, 97.5% C (original)	min, max C (imputed)	2.5, 97.5% D (original)	min, max D (imputed)	2.5, 97.5% E (original)	min, max E (imputed)	N (original)	N (imputed)
focal groups	11	-85.36, 199.34	18.13, 91.17	1464.52, 1490.92	1473.21, 1481.68	248.41, 299.11	263.7, 279.77	944	993
	15	-146.84, 405.73	111.29, 230.31	1350.26, 1410.9	1371.37, 1383.98	205.44, 313.68	255.63, 277.99	148	157
	17	-360.62, 895.66	127.91, 304.03	1365.9, 1432.07	1390.11, 1401.16	151.27, 465.8	274.5, 319.04	172	184
	1B	248.01, 490.97	340.23, 409.23	1778.1, 1801.6	1788.56, 1791.46	272.45, 322.36	292.12, 304.2	2058	2094
	1H	-319.03, 83.95	-203.16, -72.7	1417.03, 1446.43	1428.43, 1433.59	216.52, 272.17	233.99, 247.26	523	539
	1N	-803.82, 1309.86	181.9, 465.59	1666.76, 1793.93	1727.69, 1738.25	107.87, 381.26	238.84, 265.57	97	100
	2	-1279.04, -222.36	-703.7, -440.76	1563.37, 1594.58	1581.35, 1589.46	148.23, 204.3	179.34, 196.26	1127	1159
	26	-422.17, 1485.79	86.76, 759.54	1523.84, 1756.82	1590.86, 1684.84	-108.46, 884.94	239.86, 576.36	23	27
	4B	-804.02, 57.97	-417.45, -318.09	1353.23, 1403.16	1375.58, 1387.55	175.83, 263.61	215.87, 227.67	260	278
	7A	-2233.17, -283.9	-1351.99, -1059.39	1348.26, 1425.66	1375.84, 1396.52	116.81, 201.55	154.47, 170.36	85	91

rival groups	11	-48.21, 218.66	46.13, 128.77	1476.46, 1503.02	1485.33, 1493.15	263.3, 318.81	282.4, 297.68	932	971
	12	161.7, 1028.98	559.97, 588.38	1404.38, 1485.49	1441.29, 1446.86	165.44, 476.66	308.94, 317.98	53	55
	15	-17.07, 610.41	306.03, 530.64	1360.08, 1457.23	1417.48, 1445.54	232.05, 459.06	353.22, 508.54	43	47
	17	-600.68, 732.85	-1.86, 167.69	1385.87, 1464.51	1418.59, 1431.34	164.6, 434.43	282.52, 319.09	96	102
	18	-374.28, 1469.62	465.4, 547.23	1386.32, 1725.2	1555.06, 1555.75	-68.16, 755.38	310.82, 343.41	7	8
	1B	95.93, 353.31	213.72, 251.04	1782.37, 1806.74	1793.74, 1796.76	256.21, 302.62	277.45, 283.79	2224	2244
	1H	-362, 130.4	-175.09, -95.41	1391.04, 1426.55	1403.98, 1411.24	210.27, 278.27	232.48, 245.97	449	468
	1K	-281.45, 747.42	166.67, 197.02	1368.82, 1546.11	1425, 1440.52	161.74, 405.99	255.73, 268.48	37	39
	1N	-688.06, 1135.92	145.35, 306.7	1669.13, 1742.8	1704.17, 1708.51	134.46, 324.48	221.18, 237.88	167	168
	2	574.86, 855.36	703.15, 761.46	1651.62, 1718.46	1685.67, 1704.45	345.22, 530.76	431.09, 479.67	816	862
	4B	-438.89, 131.01	-192.36, -118.55	1342.29, 1383.2	1358.47, 1369.81	198.08, 275.34	228.47, 241.72	369	394
	7A	-354.21, 333.39	-173.58, 209.83	1407.37, 1486.34	1435.67, 1467.18	214.36, 382.66	260.25, 349.56	110	123

TABLE S6. CONTEST SUCCESS FOR EACH GROUP

Each group in the final dataset (N = 90 contests across 11 groups) is represented by a unique row. Columns show the group ID, the number of contests in which that group participated, the number of contests the group won, and the proportion of contests won (N won / N participated). Rows are ranked in decreasing order of proportion of contests won.

Group	N participated	N won	Proportion won
1B	50	43	0.86
1H	23	14	0.61
2	10	5	0.50
11	51	21	0.41
4B	13	4	0.31
17	12	2	0.17
7A	9	1	0.11
15	9	0	0
1K	1	0	0
30	1	0	0
26	1	0	0

TABLE S7. MODELS WITHOUT 5,000-MODEL CUTOFF

Models presented are those in the top model set, without removing models that did not appear in > 5,000 iterations of the random sampling procedure (see Materials and Methods). Null model (including only random effects of focal and rival group identity) AICc = 102.0 (ΔAICc from median value of best-fit model = 21.5). Models that are also included in Table S2 are marked with an asterisk in the "N models" column.

**Predictors**	**N models**	**Median AICc (2.5%, 97.5%)**	**Median l_i (2.5%, 97.5%)**	**Median w_i (2.5%, 97.5%)**
# males, max male age, mean male weight, # males : max male age	119	80.471 (80.045, 80.882)	1.000 (1.000, 1.000)	0.272 (0.234, 0.301)
# males, max male age, mean male weight	10000*	80.826 (80.294, 81.328)	1.000 (1.000, 1.000)	0.345 (0.267, 0.414)
# males, max male age, max male weight, # males : max male age	9999*	82.547 (81.434, 83.452)	0.426 (0.279, 0.707)	0.146 (0.104, 0.208)
# members, max member age, mean member weight	10000*	82.631 (82.247, 82.999)	0.405 (0.339, 0.483)	0.140 (0.109, 0.163)
# males, max male age, max male weight	10000*	82.945 (82.038, 83.665)	0.347 (0.247, 0.508)	0.120 (0.091, 0.150)
# members, max member age, max member weight, # members : max member age, # members : max member weight	44	83.351 (81.720, 84.997)	0.260 (0.143, 0.634)	0.078 (0.046, 0.138)
# members, max member age, max member weight, # members : max member weight	12	84.077 (82.202, 85.013)	0.193 (0.136, 0.538)	0.055 (0.043, 0.095)
# members, max member age, max member weight, # members : max member age	1630	84.296 (82.930, 85.626)	0.178 (0.092, 0.340)	0.057 (0.033, 0.088)
# males, max male age, # males : max male age	10000*	84.635 (84.635, 84.635)	0.149 (0.114, 0.191)	0.052 (0.037, 0.062)

# members, max member age, max member weight	9998*	84.700 (83.434, 85.886)	0.144 (0.077, 0.269)	0.050 (0.029, 0.078)
# males, max male age	10000*	84.867 (84.867, 84.867)	0.133 (0.101, 0.170)	0.046 (0.033, 0.055)
# members, mean member weight	10000*	85.354 (84.922, 85.758)	0.104 (0.086, 0.125)	0.036 (0.028, 0.042)
# members, mean member age, max member weight	7046*	86.268 (85.164, 86.935)	0.066 (0.051, 0.115)	0.022 (0.017, 0.034)
# members, max member age, # members : max member age	9620*	86.338 (86.338, 86.338)	0.064 (0.052, 0.082)	0.022 (0.017, 0.027)
# members, max member weight	4221	86.408 (85.152, 87.120)	0.062 (0.050, 0.112)	0.020 (0.016, 0.032)
# members, mean member age, max member weight	1828	87.062 (87.062, 87.062)	0.053 (0.050, 0.062)	0.017 (0.014, 0.019)
# members, max member age	1069	87.151 (87.151, 87.151)	0.052 (0.050, 0.061)	0.016 (0.013, 0.019)

TABLE S8-S14: REPORTING OF MODEL OUTPUT

In all tables, significant fixed effects are highlighted with bold font.

Table S8: Results of model predicting individual weight from sex. N = 77,023 weight measurements, 1651 unique males, 1430 unique females.

Fixed effect	β	SE	χ^2 (df)	P
Intercept	1198.67	9.36		
**Sex (male)**	108.74	11.89	**80.15**	**< 0.01**

Random effect	Variance	SD
Individual ID	26619	163.2

Table S9: Results of model predicting individual head width from sex. N = 11,284 head width measurements, 1360 unique males, 1094 unique females.

Fixed effect	β	SE	χ^2 (df)	P
Intercept	34.68	0.18		
**Sex (male)**	0.75	0.23	**10.43**	**< 0.01**

Random effect	Variance	SD
Individual ID	27.91	5.28

Table S10: Results of model predicting the number of intergroup contests a male has participated in during his lifetime by his age. N = 319 contests, 853 unique males. The fixed effect of male age is scaled with a mean of zero and unit variance. In random effect table, OLRE = observation level random effect.

Fixed effect	β	SE	χ^2 (df)	P
Intercept	1.11	0.18		
**Age**	0.83	0.03	**507.7**	**< 0.01**

Random effect	Variance	SD
OLRE	0.47	0.68
Group	0.52	0.73

Table S11: results of model predicting the likelihood of intergroup contest success from senior male age. N = 90 contests, 23 unique senior males. The fixed effect of senior male age is scaled with a mean of zero and unit variance.

**Fixed effect**	**β**	**SE**	**χ^2 (df)**	**P**
Intercept	1.65	0.33		
**Senior male age**	-1.19	0.34	**11.34**	**< 0.01**

**Random effect**	**Variance**	**SD**
Senior male ID	0.28	0.53

Table S12: results of model predicting the likelihood of male eviction from male age. N = 57 eviction events, 148 evicted males, 543 total males. The fixed effect of male age is scaled with a mean of zero and unit variance.

**Fixed effect**	**β**	**SE**	**χ^2 (df)**	**P**
Intercept	-3.47	0.51		
**Male age**	0.49	0.14	**15.7**	**< 0.01**

**Random effect**	**Variance**	**SD**
Male ID	1.64	1.28
Eviction event ID	5.83	2.42
Group ID	0.00	0.00

Table S13: results of model predicting the proportion of pups in litters that were sired by within-group senior males from senior male age. N = 133 unique litters, 46 unique senior males. The fixed effect of senior male age is scaled with a mean of zero and unit variance.

**Fixed effect**	**β**	**SE**	**χ^2 (df)**	**P**
Intercept	-0.19	0.15		
**Senior male age**	-0.32	0.11	**9.05**	**< 0.01**

**Random effect**	**Variance**	**SD**
Litter ID	0.44	0.66
Senior male ID	0.00	0.00
Group ID	0.09	0.30

Table S14: results of model predicting the proportion of pups in litters that were sired by senior males in any group (i.e., including extra-group paternity) from senior male age. N = 145 unique litters, 50 unique senior males. The fixed effect of senior male age is scaled with a mean of zero and unit variance.

**Fixed effect**	**β**	**SE**	**χ^2 (df)**	**P**
Intercept	-0.14	0.18		
Senior male age	-0.17	0.11	2.22	0.14

**Random effect**	**Variance**	**SD**
Litter ID	0.48	0.69
Senior male ID	0.04	0.20
Group ID	0.16	0.39

Table S15: Comparison of best-fit model estimates from original dataset (N = 90) to best-fit model estimates using a dataset with only contests that had one senior male in either group (N = 71).

**Predictor**	**Original datasets estimate median (2.5%, 97.5%)**	**Dataset w/ one senior male estimate (SE)**
# males	1.76 (1.74, 1.78)	1.75 (0.50)
max male age	1.22 (1.20, 1.24)	1.26 (0.55)
mean male weight	1.38 (1.31, 1.45)	1.38 (0.45)

SUPPLEMENTARY INFORMATION REFERENCES

1. M. Dyble, T. M. Houslay, M. B. Manser, T. Clutton-Brock, Intergroup aggression in meerkats. *Proceedings of the Royal Society B: Biological Sciences* **286** (2019).
2. A. J. Wilson, *et al.*, Indirect genetics effects and evolutionary constraint: An analysis of social dominance in red deer, *Cervus elaphus*. *Journal of Evolutionary Biology* **24**, 772–783 (2011).
3. S. M. Lane, A. J. Wilson, M. Briffa, Analysis of direct and indirect genetic effects in fighting sea anemones. *Behavioral Ecology* **31**, 540–547 (2020).
4. J. D. Hadfield, MCMC methods for multi-response Generalized Linear Mixed Models: The MCMCglmm R package. *Journal of Statistical Software* **33** (2010).
5. R. A. Bradley, M. E. Terry, Rank analysis of incomplete block designs: I. the method of paired comparisons. *Biometrika* **39**, 324–345 (1952).
6. H. J. Nichols, W. Amos, M. A. Cant, M. B. V. Bell, S. J. Hodge, Top males gain high reproductive success by guarding more successful females in a cooperatively breeding mongoose. *Animal Behaviour* **80**, 649–657 (2010).
7. J. L. Sanderson, J. Wang, E. I. K. Vitikainen, M. A. Cant, H. J. Nichols, Banded mongooses avoid inbreeding when mating with members of the same natal group. *Molecular Ecology* **24**, 3738–3751 (2015).
8. D. A. Wells, M. A. Cant, H. J. Nichols, J. I. Hoffman, A high-quality pedigree and genetic markers both reveal inbreeding depression for quality but not survival in a cooperative mammal. *Molecular Ecology* **27**, 2271–2288 (2018).
9. F. J. Thompson, H. H. Marshall, E. I. K. Vitikainen, M. A. Cant, Causes and consequences of intergroup conflict in cooperative banded mongooses. *Animal Behaviour* **126**, 31–40 (2017).
10. M. A. Cant, E. Otali, F. Mwanguhya, Fighting and mating between groups in a cooperatively breeding mammal, the banded mongoose. *Ethology* **108**, 541–555 (2002).
11. S. van Belle, C. J. Scarry, Individual participation in intergroup contests is mediated by numerical assessment strategies in black howler and tufted capuchin

monkeys. *Philosophical Transactions of the Royal Society B: Biological Sciences* **370** (2015).

12. K. A. Cassidy, D. R. MacNulty, D. R. Stahler, D. W. Smith, L. D. Mech, Group composition effects on aggressive interpack interactions of gray wolves in Yellowstone National Park. *Behavioral Ecology* **26**, 1352–1360 (2015).
13. S. Perry, Intergroup encounters in wild white-faced capuchins (*Cebus capucinus*). *International Journal of Primatology* **17** (1996).
14. M. C. Crofoot, I. C. Gilby, M. C. Wikelski, R. W. Kays, Interaction location outweighs the competitive advantage of numerical superiority in *Cebus capucinus* intergroup contests. *Proceedings of the National Academy of Sciences of the United States of America* **105**, 577–581 (2008).
15. A. M. Roth, M. Cords, Effects of group size and contest location on the outcome and intensity of intergroup contests in wild blue monkeys. *Animal Behaviour* **113**, 49–58 (2016).
16. T. J. M. Arseneau-Robar, *et al.*, Female monkeys use both the carrot and the stick to promote male participation in intergroup fights. *Proceedings of the Royal Society B: Biological Sciences* **283** (2016).
17. T. J. M. Arseneau-Robar, *et al.*, Male monkeys use punishment and coercion to de-escalate costly intergroup fights. *Proceedings of the Royal Society B: Biological Sciences* **285** (2018).
18. T. R. Harris, Multiple resource values and fighting ability measures influence intergroup conflict in guerezas (*Colobus guereza*). *Animal Behaviour* **79**, 89–98 (2010).
19. M. M. Robbins, S. C. Sawyer, Intergroup encounters in mountain gorillas of Bwindi Impenetrable National Park, Uganda. *Behaviour* **144**, 1497–1519 (2007).
20. M. O. Mirville, *et al.*, Low familiarity and similar 'group strength' between opponents increase the intensity of intergroup interactions in mountain gorillas (*Gorilla beringei beringei*). *Behavioral Ecology and Sociobiology* **72** (2018).
21. K. Cooksey, *et al.*, Socioecological Factors Influencing Intergroup Encounters in Western Lowland Gorillas (*Gorilla gorilla gorilla*). *International Journal of Primatology* **41**, 181–202 (2020).

22. H. Sugiura, *et al.*, Variation in intergroup encounters in two populations of Japanese macaques. *International Journal of Primatology* **21** (2000).

23. P. T. Mehlman, R. S. Parkhill, Intergroup interactions in wild Barbary Macaques (*Macaca sylvanus*), Ghomaran Rif Mountains, Morocco. *American Journal of Primatology* **15**, 31–44 (1988).

24. M. L. Wilson, S. M. Kahlenberg, M. Wells, R. W. Wrangham, Ecological and social factors affect the occurrence and outcomes of intergroup encounters in chimpanzees. *Animal Behaviour* **83**, 277–291 (2012).

25. A. C. Markham, S. C. Alberts, J. Altmann, Intergroup conflict: Ecological predictors of winning and consequences of defeat in a wild primate population. *Animal Behaviour* **84**, 399–403 (2012).

26. A. N. Radford, Duration and outcome of intergroup conflict influences intragroup affiliative behaviour. *Proceedings of the Royal Society B: Biological Sciences* **275**, 2787–2791 (2008).

27. F. Koch, J. Signer, P. M. Kappeler, C. Fichtel, The role of the residence-effect on the outcome of intergroup encounters in Verreaux's sifakas. *Scientific Reports* **6** (2016).

28. G. Benadi, C. Fichtel, P. Rappeler, Intergroup relations and home range use in Verreaux's sifaka (*Propithecus verreauxi*). *American Journal of Primatology* **70**, 956–965 (2008).

29. R. J. Lewis, A. A. Sandel, S. Hilty, S. E. Barnett, The collective action problem but not numerical superiority explains success in intergroup encounters in Verreaux's sifaka (*Propithecus verreauxi*): implications for individual participation and free-riding. *International Journal of Primatology* **41**, 305–324 (2020).

MOVIE S1 LEGEND

Banded mongoose intergroup contests. Banded mongoose intergroup contests often begin with groups assuming "battle line" formations. This can be followed by potentially-violent one-on-one fights between opposing group members, or scenarios where multiple members from one group gang up on single individuals from the rival group.
